# Occurrence and Treatment of Antibiotic-Resistant Bacteria Present in Surface Water

**DOI:** 10.3390/membranes13040425

**Published:** 2023-04-11

**Authors:** João Sério, Ana Paula Marques, Rosa Huertas, João Goulão Crespo, Vanessa Jorge Pereira

**Affiliations:** 1 iBET—Instituto de Biologia Experimental e Tecnológica, Apartado 12, 2780-901 Oeiras, Portugal; 2 Instituto de Tecnologia Química e Biológica António Xavier, Universidade Nova de Lisboa, Av. da República, 2780-157 Oeiras, Portugal; 3 LAQV-REQUIMTE, Department of Chemistry, NOVA School of Science and Technology, Universidade NOVA de Lisboa, 2829-516 Caparica, Portugal

**Keywords:** antibiotic-resistant bacteria, surface water treatment, membrane filtration, photolysis, photocatalytic membrane reactor

## Abstract

According to the World Health Organization, antibiotic resistance is one of the main threats to global health. The excessive use of several antibiotics has led to the widespread distribution of antibiotic-resistant bacteria and antibiotic resistance genes in various environment matrices, including surface water. In this study, total coliforms, *Escherichia coli* and enterococci, as well as total coliforms and *Escherichia coli* resistant to ciprofloxacin, levofloxacin, ampicillin, streptomycin, and imipenem, were monitored in several surface water sampling events. A hybrid reactor was used to test the efficiency of membrane filtration, direct photolysis (using UV-C light emitting diodes that emit light at 265 nm and UV-C low pressure mercury lamps that emit light at 254 nm), and the combination of both processes to ensure the retention and inactivation of total coliforms and *Escherichia coli* as well as antibiotic-resistant bacteria (total coliforms and *Escherichia coli*) present in river water at occurrence levels. The membranes used (unmodified silicon carbide membranes and the same membrane modified with a photocatalytic layer) effectively retained the target bacteria. Direct photolysis using low-pressure mercury lamps and light-emitting diode panels (emitting at 265 nm) achieved extremely high levels of inactivation of the target bacteria. The combined treatment (unmodified and modified photocatalytic surfaces in combination with UV-C and UV-A light sources) successfully retained the bacteria and treated the feed after 1 h of treatment. The hybrid treatment proposed is a promising approach to use as point-of-use treatment by isolated populations or when conventional systems and electricity fail due to natural disasters or war. Furthermore, the effective treatment obtained when the combined system was used with UV-A light sources indicates that the process may be a promising approach to guarantee water disinfection using natural sunlight.

## 1. Introduction

Good water quality is essential to human health. However, environmental degradation hinders the sufficient and safe distribution of water worldwide. The disposal of residues resulting from human practices, hospital facilities, agricultural activities, and industries without appropriate treatment have significantly compromised aquatic ecosystems.

Antimicrobial resistance is a hidden but expanding threat that may also be disseminated by the continuous pollution of many environment matrices, such as water, sludge, and soil [1,2,3]. Over the last decades, treatable infections have become lethal, resulting in higher mortality rates, lengthy hospital stays, and increased healthcare costs [4]. The World Health Organization (WHO) has declared that antimicrobial resistance is one of the top 10 global public threats facing humanity [5]. It is estimated that antimicrobial resistance-related diseases currently lead to 700,000 deaths per year and that they could lead to 10 million deaths by 2050 [6,7]. The spread of COVID-19 around the globe led to an increased consumption of antibiotics to prevent bacterial superinfections [8]. A recent study highlighted the devastating consequences for antimicrobial resistance management associated with inappropriate antibiotic use by COVID-19 patients [9]. Antibiotic-resistant bacteria and antibiotic-resistant genes have proliferated extensively due to the misuse and overuse of antibiotics and the increasing concentration of pharmaceuticals in the environment, constituting an aggravated risk of epidemics [3,10]. Even carbapenems, a group of powerful last-resort antibiotics administered in cases of severe infection, are gradually losing their effectiveness [4,11]. In addition, multidrug-resistant organisms are spreading around the globe in the context of healthcare and foreign travel. They can also reportedly be transmitted to humans from wild animals, livestock, and pets [12]. When the degradation of these emerging compounds is inadequate and poorly treated effluent is discharged, they end up entering rivers, reservoirs, estuaries, groundwater, and drinking water supplies [13,14,15,16]. Currently, more than 200 different types of pharmaceutical compounds have been detected in various rivers worldwide, coming directly from wastewater treatment plants effluent [16].

Membrane filtration is a proven alternative to achieve high water quality by selectively removing pollutants and microorganisms of various sizes [17]. Filtration methods, such as microfiltration and ultrafiltration, may be efficiently used to remove pathogens, especially bacteria, fungi, and protozoa [18]. Silicon carbide membranes have been developed due to their high chemical, thermal, and mechanical resistance [19]. In addition, silicon carbide membranes exhibit high permeability, controlled porosity, and a smooth top layer, and are efficient in removing indicator bacteria and suspended solids, as well as oil and grease, from wastewater matrices [20,21,22,23]. Modifying membranes by depositing photocatalytic nanoparticles can change their properties and ultimately increase their photocatalytic potential (conferring a self-cleaning property). This makes it possible to treat various contaminated water sources and reduce fouling, the major drawback of membrane processes [17,24]. Sosa et al. [25] demonstrated that a combined treatment using silicon carbide membranes modified with zinc oxide was, when exposed to UV-A LEDs that emit at 365 nm, very efficient at inactivating total coliforms and *Escherichia coli* present in surface water at occurrence levels. In this study, new photocatalytic silicon carbide membranes were developed using a previously described solvent-free sol-gel method [23] in which the titanium dioxide photocatalytic coatings are deposited at low temperatures using only aqueous solutions. These membranes are expected to retain microorganisms and contribute to their inactivation by photocatalysis. Due to its large bandgap, the application of titanium dioxide is limited to the UV range (<390 nm), where only a very small fraction of the total solar radiation is used [16]. Since the modified membranes are intended to be activated by sunlight, the titanium dioxide nanoparticles were doped with copper to reduce their bandgap energy by creating defects in their structure, which increases the optical absorption in the visible range. In addition, copper acts as an active trap of electrons, reducing the rate of electron-hole recombination [24,26]. Mahmud et al. [27] reported that this doping allowed titanium dioxide to degrade methylene blue under simulated sunlight conditions. Furthermore, Pongwan et al. [28] reported a shift in the band gap energy of titanium dioxide to 2.83 eV after doping with copper, which enabled the mineralization of oxalic acid and formic acid under visible light irradiation. Moreover, Dunlop et al. [29] reported the inactivation of clinically relevant pathogens by photocatalytic coatings.

Ultraviolet radiation (UV) using low- or medium-pressure mercury lamps is effective at inactivating a wide range of waterborne pathogens and is commonly used in many water and wastewater treatment plants [30]. UV light-emitting diodes (LEDs) have emerged as a promising alternative treatment [31,32,33,34,35,36]. LEDs do not produce mercury waste; are more compact, durable, and energy efficient; do not require a stabilization time; and can emit light at different wavelengths [31,32,33,34,35,36].

Hybrid treatment systems combining various technologies such as membrane filtration, ultraviolet irradiation, and photocatalysis have reportedly shown promising results in microbial retention, disinfection, and degradation of contaminants [21,22,25,37,38,39]. Previous laboratory-scale studies combining light-emitting diodes (LEDs) and photocatalytic silicon carbide membranes have achieved promising results in terms of inactivation (>2.5-log inactivation using LEDs emitting at 255 and 265 nm) and retention (>96%) of quality indicator bacteria present in real wastewater and surface water matrixes, as well as further production of a reusable high-quality permeate [21,22]. The combined treatment was tested in this study for the retention and inactivation of antibiotic-resistant bacteria present at occurrence levels in surface water using a submerged photocatalytic membrane reactor previously assembled [40] that treats a large volume of water (10 L), using modified and unmodified silicon carbide membranes, custom-made LED panels that emit light at 265 and 385 nm, and low-pressure mercury lamps (LP-UV) that emit light at 254 nm.

This work focused on testing the occurrence of water quality indicator bacteria (total coliforms, *E. coli* and enterococci) as well as total coliforms and *E. coli* resistant to various antibiotics (ciprofloxacin, levofloxacin, imipenem, ampicillin, and streptomycin) in surface water. The treatment efficiency of total coliforms and *E. coli*, as well as antibiotic-resistant total coliforms and *E. coli*, present at occurrence levels in river water was then tested using membrane filtration (with unmodified membranes and membranes modified with titanium dioxide and copper), direct UV photolysis, and the combination of both processes in a hybrid reactor.

## 2. Materials and Methods

### 2.1. Surface Water Matrix

Surface water samples were collected from the Tagus River (Algés, Portugal) in sterile glass containers between January 2020 and August 2021. Field and travel blanks were also analyzed and were free from contamination by the target microorganisms.

The water samples were characterized in terms of pH (Crison MicropH 2002) as well as total solids (TS), total suspended solids (TSS), and total dissolved solids (TDS) (Standard Method 2540) [41] to assess their overall quality.

A microbiological characterization of the samples in terms of total coliforms, *E. coli*, and enterococci—bacteria frequently used as microbiological indicators of water quality [42,43]—was performed. The analysis of antibiotic-resistant bacteria was also performed as detailed below.

#### 2.1.1. Total coliforms and *Escherichia coli*

The water samples were analyzed in terms of the occurrence of total coliforms and *E. coli* using the Colilert-18 kit (IDEXX, Maine, ME, USA) described by Warden et al. [44,45,46]. Briefly, 100 mL of each water sample (undiluted or diluted) was added to a sterile flask, and the substrate was added and shaken until a homogenized solution was obtained. The liquid was then transferred to a Quanti-Tray 2000 and incubated at 35 ± 0.5 °C for 18 h. When coliforms metabolize O-nitrophenyl-beta-D-galactopyranoside (ONPG), the sample turns yellow. When *E. coli* metabolizes 4-methyl-umbelliferyl-β-D-glucuronide (MUG), the sample also fluoresces. The yellow wells were counted to determine the MPN/100 mL of total coliforms, and the yellow/fluorescent wells were counted using a BLAK-RAY^®^ lamp (model UVL-21) that emits at 360 nm to determine the MPN/100 mL of *E. coli*.

The characterization of antibiotic-resistant bacteria was performed following a modified protocol of the Colilert-18 kit previously described by Galvin et al. [47]. The procedure follows the same main steps; however, before mixing the substrate with the samples, a specific concentration of the chosen antibiotic was added to the sample. By the end of the incubation, only resistant bacteria would result in color and fluorescent change of the Quanti-tray wells. The target antibiotics (ciprofloxacin, levofloxacin, imipenem, ampicillin, and streptomycin) belong to four different classes and were selected based on data resulting from the surveillance of antimicrobial resistance in Europe 2018 [48,49,50]. Following addition of the antibiotic (described in Appendix A), the sample was processed in Colilert-18 Quanti-Trays according to the manufacturer’s instructions. After incubation at 35 ± 0.5 °C for 18 h, the positive wells were counted following the manufacturer’s instructions.

#### 2.1.2. Enterococci

The surface water samples were also analyzed in terms of the occurrence of intestinal enterococci such as *Enterococcus faecium* and *E. faecalis* using an Enterolert-E Kit (IDEXX, Maine, ME, USA), as described in the literature [44,51,52]. The procedure is similar to the before mentioned Colilert-18 kit. After the specific substrate was mixed with the samples, the samples were incubated for 24 h at 41 ± 0.5 °C. The positive wells were determined using a BLAK-RAY^®^ lamp (model UVL-21) that emits at 360 nm to read the fluorescence emission, which indicates the metabolization of the substrate 4-methyl-umbelliferyl-β-D-glucoside by β-glucosidase.

### 2.2. Surface Water Treatment Experiments

A submerged hybrid reactor described in detail in previous publications [22,40] was used to test the effectiveness of membrane filtration, photolysis, and the combination of both processes to treat total coliforms, *E. coli*, as well as antibiotic-resistant bacteria (total coliforms and *E. coli*) present at occurrence levels in surface water. The membrane is placed in the center of the submerged photocatalytic membrane reactor. The submerged flat sheet membranes (17 cm × 10 cm × 0.6 cm; LiqTech International, Ballerup, Denmark) filter from the outside to the inside. Two diaphragm pumps (12 V 3.0 A, 5.5bar; SZY-4155, Shui Zhi Yuan) were used to ensure a filtration pressure of 0.2 bar. The mixing of the system was ensured by aeration. The photocatalytic layer of the membrane is activated using LED panels that emit light at 385 nm placed at 1.6 cm from each side of the membrane. The modified membranes can therefore be easily irradiated, which decreases fouling and increases the feed treatment due to direct and indirect photolysis.

**Figure A1:**
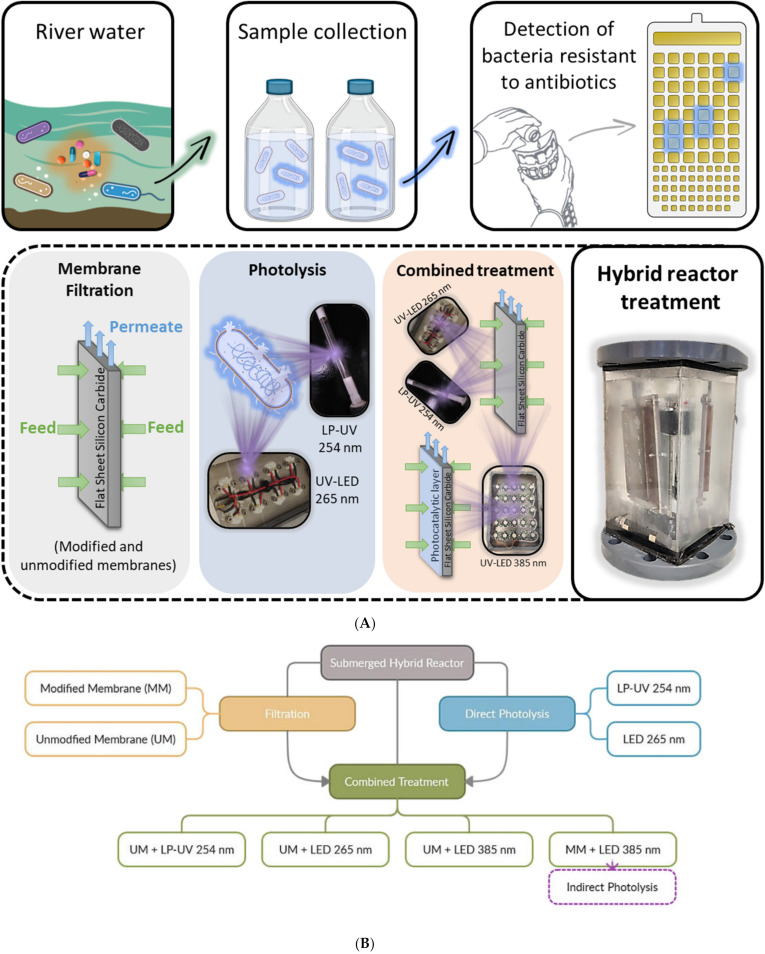
Graphical abstract (**A**) and diagram of the experiments conducted in the submerged hybrid reactor (**B**).

The experiments conducted (depicted in the Appendix A; Figure A1) included:

(1) Membrane filtration tests without the light sources using silicon carbide ceramic unmodified (UM) and modified membranes (MM) with titanium dioxide and copper previously described by Marques et al. [22]. The deposition of photocatalytic layers did not significantly affect the estimated porous properties of the modified membranes (for the unmodified and modified membrane the mean pore areas were 0.025 and 0.033 µm^2^, the average Feret diameters were 0.14 and 0.17 μm, and the average pore density values were 2.62 and 1.98 µm^−2^, respectively); The membranes used were highly hydrophilic, since a stable water contact angle was impossible to measure.
(2) Direct photolysis inactivation tests, without membrane filtration, using the different light sources. Two commercial low-pressure mercury lamps were tested (Puro TAP, UVC, 11 W, type GPH212T5L, Christchurch, New Zealand), cylindrical in shape, with a diameter of 15 mm and a length of 212 mm. Two custom-built LED panels were also tested: two panels (to place on each side of the membrane) with 8 LEDs each that emit light at 265 nm with an average irradiance of 15.33 µW/cm^2^.The panels emitting at 265 nm were custom-built for inactivation by direct photolysis.
(3) Combined treatment tests with membrane filtration (using the unmodified and modified membranes) and the different light sources (low-pressure mercury lamps, LEDs that emit light at 265 nm and LED panels that emit light at 385 nm) to evaluate direct and indirect photolysis. The two panels with 25 LEDs each that emit light at 385 nm with an average irradiance of 313.18 µW/cm^2^ were built to test the activation of photocatalytic coatings (indirect photolysis).

Before each treatment experiment, the reactor was cleaned with 70% ethanol and sterile distilled water. Initial reactor contamination was determined after 10 L of sterile distilled water had been circulated in the system for 30 min. Then, 200 mL of water was collected and analyzed for total coliforms and *E. coli* using the methods described in Section 2.1.1. All the sampling lines were free from contamination with the target microorganisms. While a homogenous starting point using spiked laboratory-grade water would have facilitated comparison across experiments, it is very important to work with real water samples that contain microorganisms at occurrence levels to evaluate the efficiency of the treatment processes under real conditions and consider the matrix effect (presence of organic matter and solids that could affect the efficiency of the UV light and filtration treatment processes). Furthermore, real water data is crucial for the development of practical water treatment technology, for both drinking water and wastewater [53]. At the beginning of the experiments, the reactor was filled with 10 liters of untreated surface water (collected the day before the experiment and stored at 4 °C for less than 18 h). It is important to test the collected water samples as soon as possible, as previous studies have shown that microorganisms lose viability over time. Different assays conducted on different days were therefore performed with different real water samples, because rather than comparing the effectiveness of the different processes, the objective of this work was to test whether the treatment solutions evaluated could be used to deal with the problem of antibiotic resistance. For a direct comparison of the different processes’ effectiveness, future studies could be conducted using a small volume of filtered real water samples spiked at the levels with the microorganisms of interest. In all tests, both the permeate and the retentate were fully recirculated. During the tests, samples of the feed and permeate were collected after 1, 10, 30, and 60 min. With the filtration system used it was not possible to differentiate between the microorganisms retained by size exclusion and adsorption. Inactivation experiments were performed to study the effects of direct photolysis on the composition of the feed using UV LED panels emitting at 265 nm (expected to be efficient to achieve inactivation due to the peak absorption of DNA) and low-pressure mercury lamps emitting at 254 nm that are widely used for disinfection.

Additional experiments were also performed combining filtration and photolysis to understand whether the total coliforms, *E. coli* and antibiotic-resistant bacteria (total coliforms and *E. coli*) retained by the membranes were inactivated by the UV light. After treatment, the membranes were gently cleaned to loosen the retentate, washed with 400 mL of sterile distilled water, and characterized with respect to total coliforms, *E. coli*, and antibiotic-resistant bacteria.

For each experiment, a dark control sample (untreated surface water sample protected from the light during the experimental time) was also analyzed for the presence of total coliform bacteria, *E. coli*, and enterococci. The obtained results show that the bacterial concentration in the dark control samples was identical to that of the untreated surface water before treatment (initial feed).

## 3. Results and Discussion

### 3.1. Characterization of the Water Matrix

Before each experiment, the collected untreated surface water was characterized in terms of pH, total solids, total suspended solids, and total dissolved solids (Table 1).

The results obtained show that the water samples collected in different months did not change considerably in terms of the neutral pH and the solids content (97% of the total solids present in the samples were dissolved).

#### Occurrence of Water Quality Indicators and Antibiotic-Resistant Bacteria

**Figure 1:**
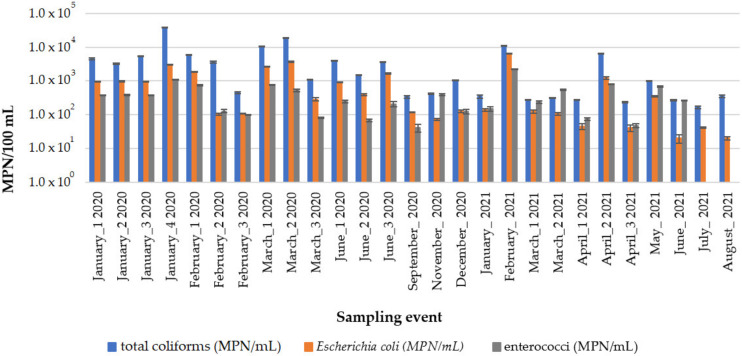
Occurrence levels (MPN/100 mL) of water quality indicator bacteria (total coliforms, *E. coli*, and enterococci) in river water.

In this study, 27 river water samples were collected in Algés, Portugal, between January 2020 and August 2021 to evaluate the occurrence levels of water quality indicator bacteria (total coliforms, *E. coli*, and enterococci). The variations in MPN/100 mL of total coliform bacteria, *E. coli*, and enterococci in the collected surface water samples are shown in Figure 1.

Figure 1 shows that the most probable number per 100 mL varied between 1.5 × 10^2^ and 1.3 × 10^4^ for total coliforms; between 2.0 × 10^1^ and 6.6 × 10^3^ for *E. coli*; and between 4.1 × 10^1^ and 2.3 × 10^3^ for enterococci. Enterococci were not analyzed in water samples collected in July and August 2021. Variations in the concentration of bacteria in the collected water samples were expected due to different pollution sources and the fact that precipitation events have been associated with increased introduction of pathogens to rivers [54]. Intestinal enterococci and *E. coli* are routinely analyzed to monitor the quality of bathing waters (e.g., EU Directive 2006/7/EC).

**Figure 2:**
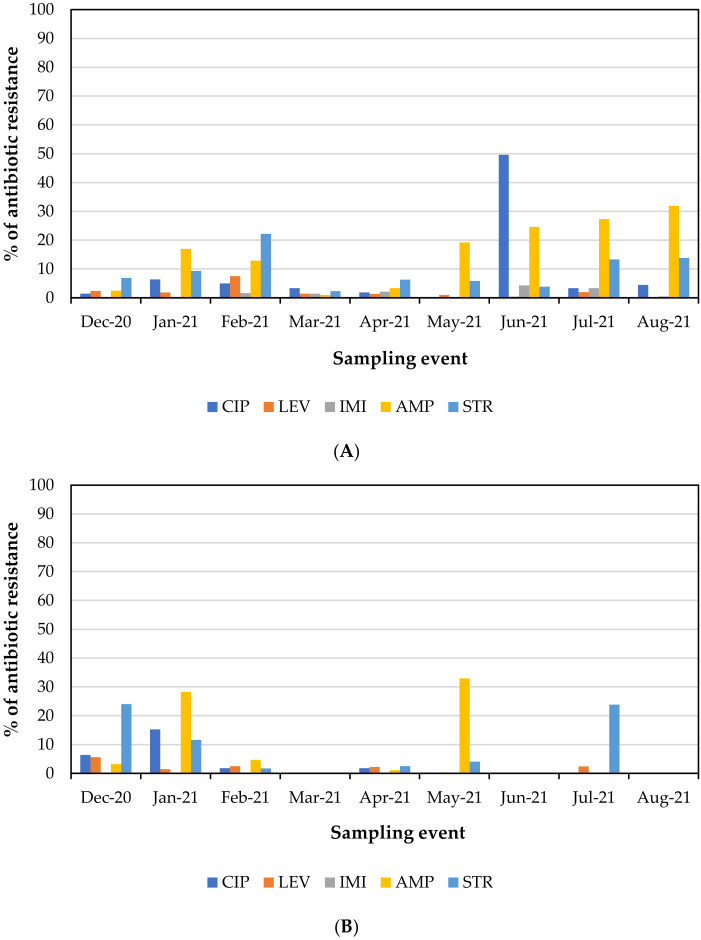
Percentage of total coliforms (**A**) and *Escherichia coli* (**B**) resistant to ciprofloxacin (CIP), levofloxacin (LEV), imipenem (IMI), ampicillin (AMP), and streptomycin (STR) present in the collected surface water samples.

**Figure A2:**
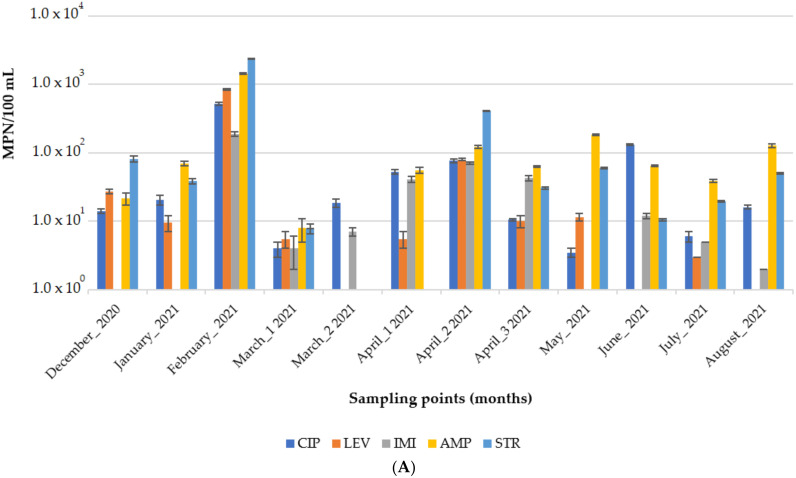

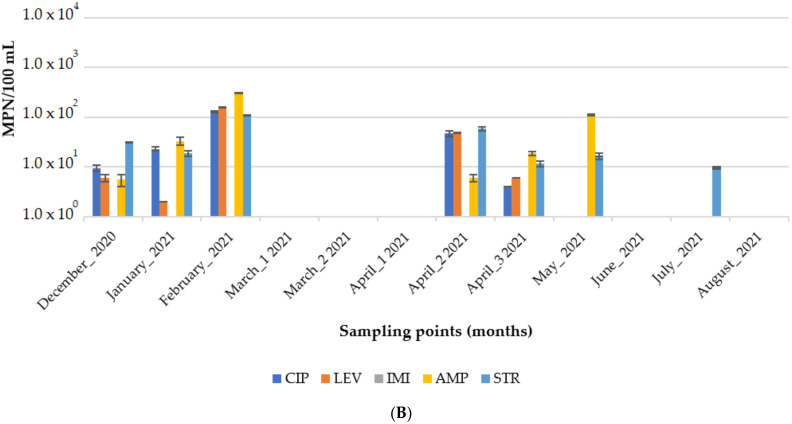
Occurrence levels (MPN/100 mL) of total coliforms (**A**) and *Escherichia coli* (**B**) resistant to ciprofloxacin (CIP), levofloxacin (LEV), imipenem (IMI), ampicillin (AMP), and streptomycin (STR) present in the collected surface water samples.

The surface water samples collected between December 2020 and August 2021 were also characterized in terms of the levels of total coliforms and *E. coli* resistant to the antibiotic’s ciprofloxacin, levofloxacin, imipenem, ampicillin, and streptomycin. The percentage of total coliforms and *E. coli* resistant to the antibiotics tested is shown in Figure 2A,B, respectively, while the occurrence levels (MPN/100 mL) are presented in Figure A2 (Appendix).

The percentage values of antibiotic-resistant bacteria fluctuated in the different sampling events due to the complexity of the matrix and the many variables that interact and lead to these changes. As mentioned in the literature, it can be hypothesized that resistance derives from the type of discharges that occurred into the river prior to sampling, inefficient treatment of effluents disposed of by treatment plants, or leaching (from landfill and sewer lines, among others) [12]. In sampling events of treated wastewater conducted by Galvin et al. [47] over fifteen days in August a similar mean percentage of antibiotic-resistant *E. coli* was reported for ampicillin, streptomycin, and ciprofloxacin.

Many of the resistant bacteria accounted for may be resistant to more than one antibiotic since the method followed to detect the resistant bacteria in a sample involved the addition of each antibiotic individually to the substrate (as detailed in Section 2.1.1).

For imipenem, a newer third-generation antibiotic used only in hospitalized patients with difficult-to-treat infections [54], low levels of resistant bacteria were detected. Since imipenem is a recent antibiotic, it was expected that resistance to its effect would not yet be widespread or developed. No imipenem-resistant *E. coli* were detected (Figure 2B). The amounts of imipenem-resistant bacteria detected in the various water river samples collected in this study were in accordance with the results described in other studies on the prevalence of carbapenem-resistant bacteria present in different types of water matrices (fountains, ponds, lakes, and rivers) [10,54,55,56,57,58].

No European legislation or guidelines exist for regulating the quality of surface water with regard to the presence of antibiotic-resistant bacteria. However, the presence of antibiotic-resistant *E. coli* has been proposed as an indicator of the presence of antibiotic-resistant bacteria and associated clinically relevant genes [57,59].

### 3.2. Water Treatment of Antibiotic-Resistant Bacteria

#### 3.2.1. Membrane Filtration Treatment

The initial flux, measured at 0.2 bar for the modified membranes with titanium dioxide and copper, was 880 ± 6 Lh^−1^m^−2^, and for the unmodified silicon carbide membranes it was 1581 ± 10 Lh^−1^m^−2^. Even though the membrane modification did not change the porous features of the modified membranes (as described above in Section 2.2), lower water production can be expected if the modified membranes are used.

**Figure 3:**
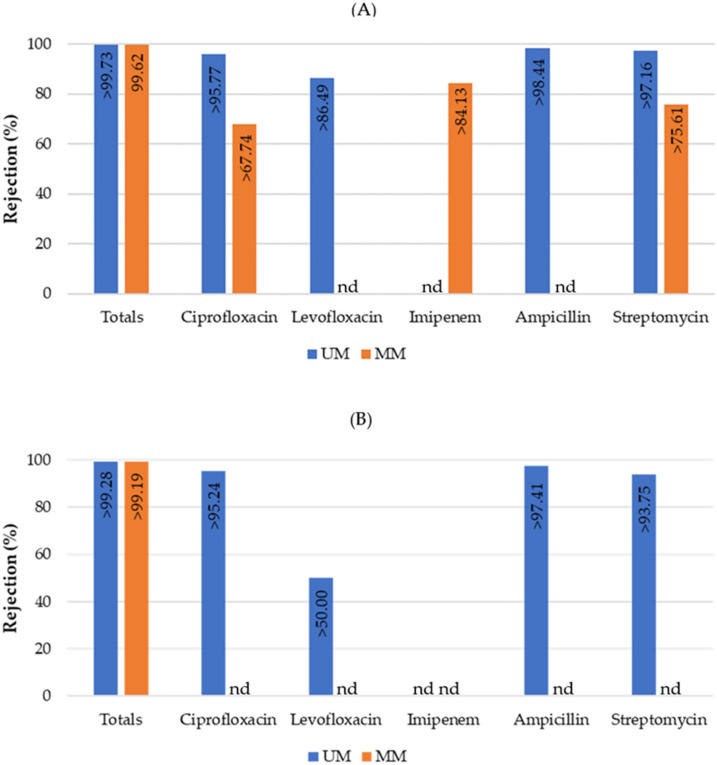
Percent rejection of total coliforms (total bars include resistant and non-resistant bacteria) and total coliforms resistant to various antibiotics (**A**) as well as *Escherichia coli* and antibiotic-resistant *Escherichia coli* (**B**) using unmodified (UM) and modified (MM) silicon carbide membranes after 60 min of filtration. The antibiotics tested were ciprofloxacin, levofloxacin, imipenem, ampicillin, and streptomycin. Columns marked “nd” indicate that the target microorganisms were not detected in the untreated surface water samples. Values marked “>” indicate that the target microorganisms were not detected in the treated water samples.

The unmodified and modified silicon carbide membranes were also tested in terms of their ability to retain total coliforms and *E. coli*, as well as total coliforms and *E. coli* resistant to various antibiotics (ciprofloxacin, levofloxacin, imipenem, ampicillin, and streptomycin) present at occurrence levels in surface water (Figure 3).

**Figure 4:**
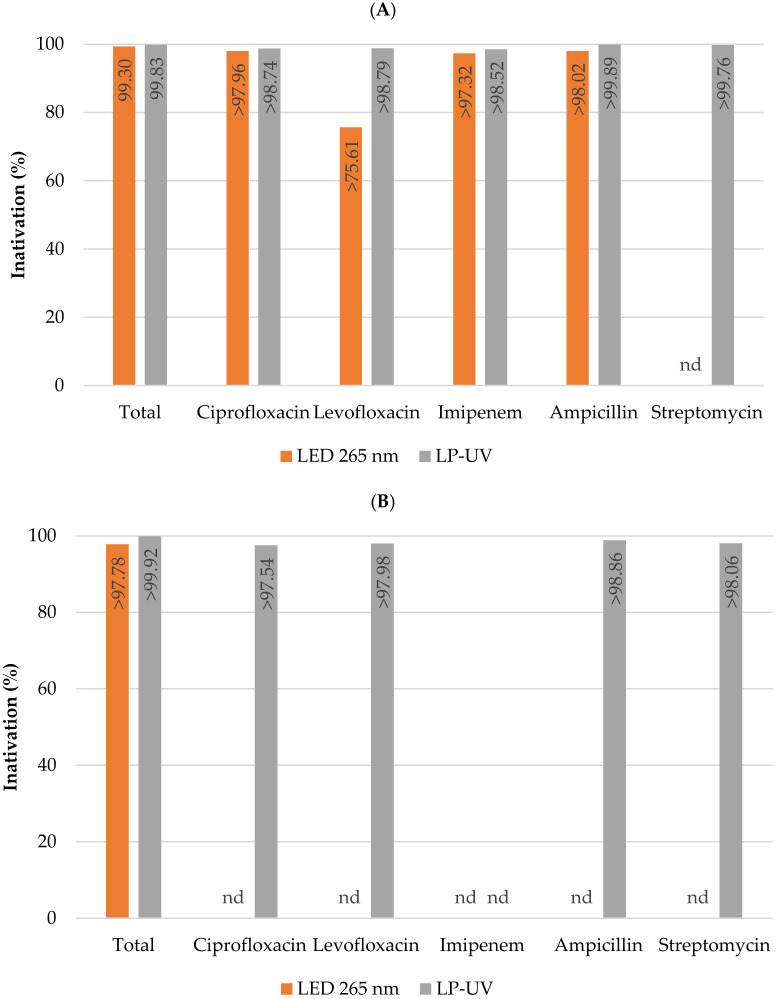
Percentage inactivation of total coliforms and total coliforms resistant to various antibiotics (**A**) as well as *Escherichia coli* and antibiotic-resistant *Escherichia coli* (**B**) by the different light sources after 60 min of treatment. The antibiotics tested were ciprofloxacin, levofloxacin, imipenem, ampicillin, and streptomycin. Columns marked “nd” indicate that the target microorganisms were not detected in the untreated surface water samples. Columns marked “>” indicate that the target microorganisms were not detected in the treated water samples after 1 h of exposure.

**Figure 5:**
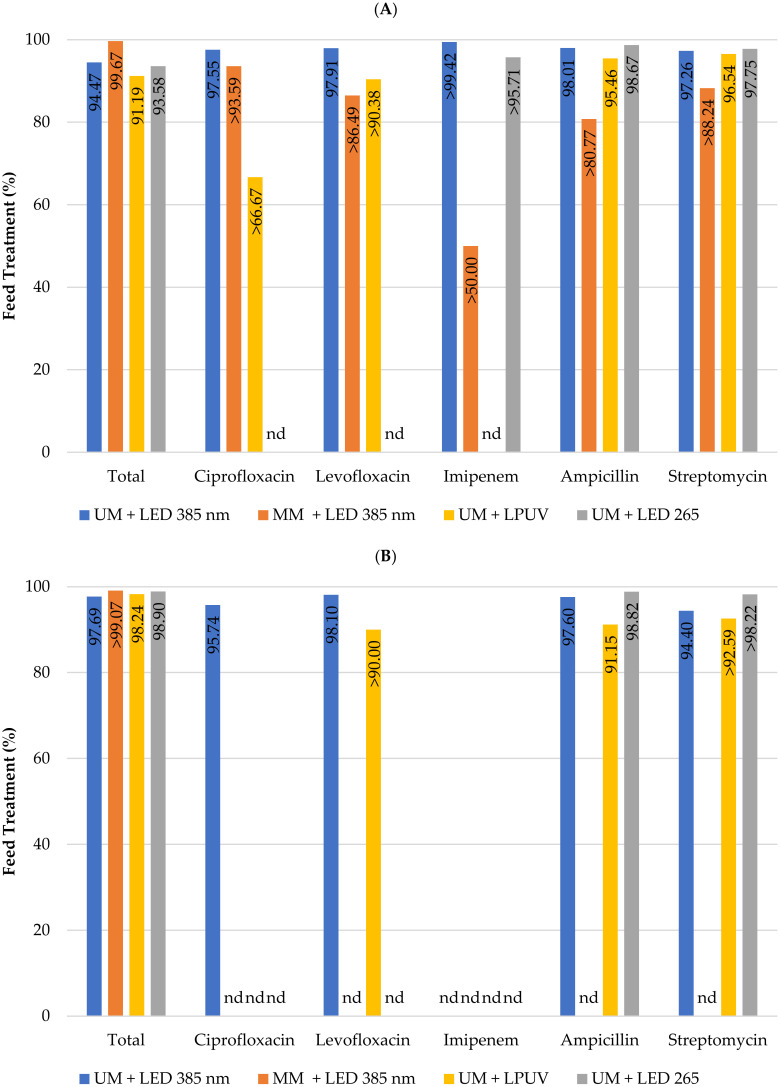
Feed treatment of total coliforms and total coliforms resistant to various antibiotics (**A**) as well as *Escherichia coli* and antibiotic-resistant *Escherichia coli* (**B**) using the hybrid reactor combined treatment. The antibiotics tested were ciprofloxacin, levofloxacin, imipenem, ampicillin, and streptomycin. Columns marked “nd” indicate that the target bacteria were not detected in the untreated surface water samples.

**Figure 6:**
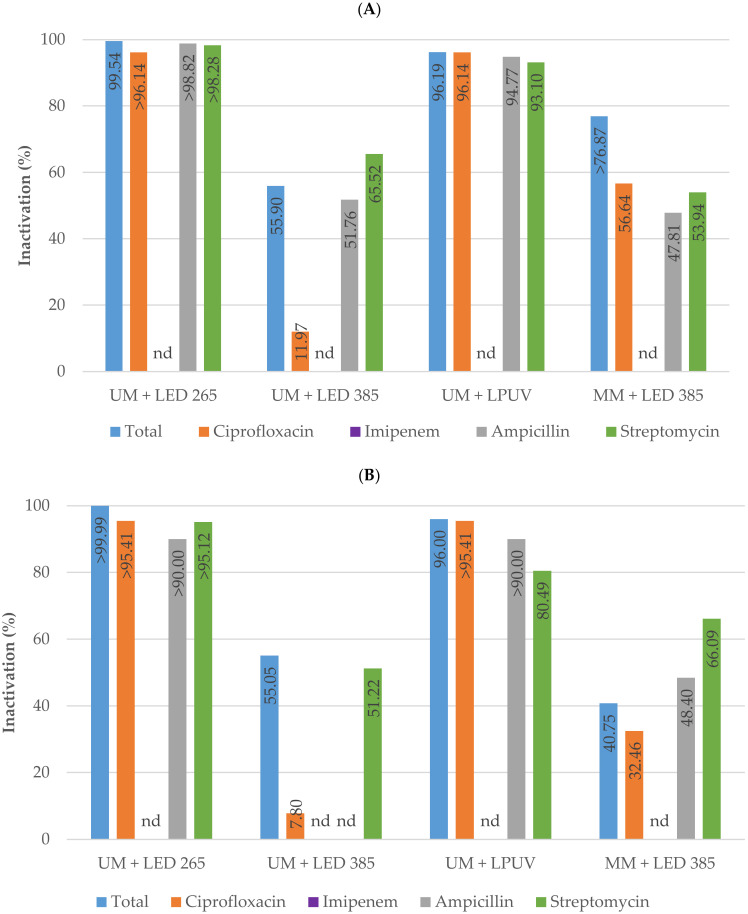
Percentage inactivation using various light sources of total coliforms and antibiotic-resistant total coliforms (**A**) as well as *Escherichia coli* and antibiotic-resistant *Escherichia coli* (**B**) adsorbed by the membranes after washing the unmodified (UM) and modified membranes (MM). The antibiotics tested were ciprofloxacin, imipenem, ampicillin, and streptomycin. Columns marked “nd” indicate that the target microorganisms were not detected in the untreated surface water samples. Columns marked “>” indicate that the target microorganisms were not detected in the treated water samples.

Since unspiked real water samples were used in these experiments, the target resistant bacteria were often not detected in the untreated water samples. The text “nd” was therefore added to the figures to highlight that the target microorganisms were not detected in the untreated surface water samples and thus the treatment performance could not be evaluated (Figure 3, Figure 4, Figure 5 and Figure 6). In addition, percentage values represented in the treatment figures with a “higher than” symbol (>) indicate that the target microorganisms were not detected in the treated water samples. The treatment percentage values shown in the figures in these cases were thus calculated based on the method detection limit and the different concentrations of the target microorganisms measured in the untreated samples.

Even though the untreated water sample collected for the filtration experiment with the modified membrane did not present antibiotic-resistant *E. coli*, the results shown in Figure 3 indicate that both membranes obtained promising values of rejection, breaching the detection limits of the methods used.

Even though different real water samples were used in the assays conducted with the modified and unmodified membranes, the levels of total coliforms, *E. coli*, and solids present in the water samples were very similar (Table A1 in Appendix A). No major differences were observed in the unmodified and modified membranes, as can be seen in Figure 3. This result was expected, since the porous features of the unmodified and modified membranes are very similar. However, the modification may prove to be important in ensuring photocatalytic activity for the feed treatment using different light sources (e.g., UV-A and solar light). Moreover, as expected, no differences in rejection were observed between the total levels of bacteria and those resistant to various antibiotics. *E. coli* are straight cylindrical rods approximately 1.1–1.5 µm (width) × 2.0–6.0 µm (length) [60] and they are effectively retained by both the unmodified and modified membranes.

**Figure A3:**
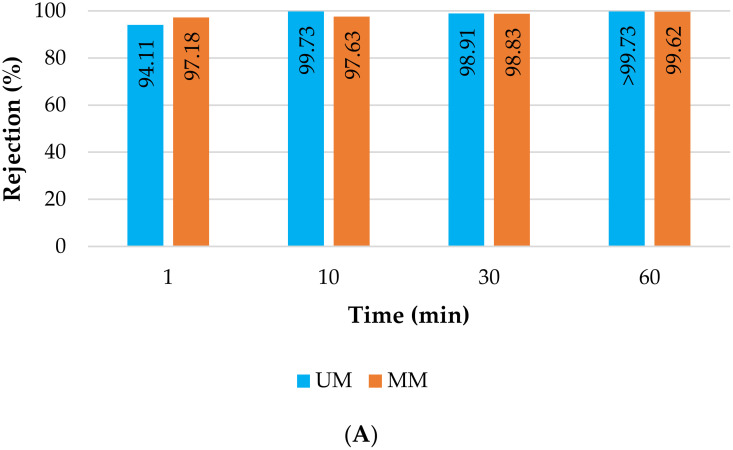

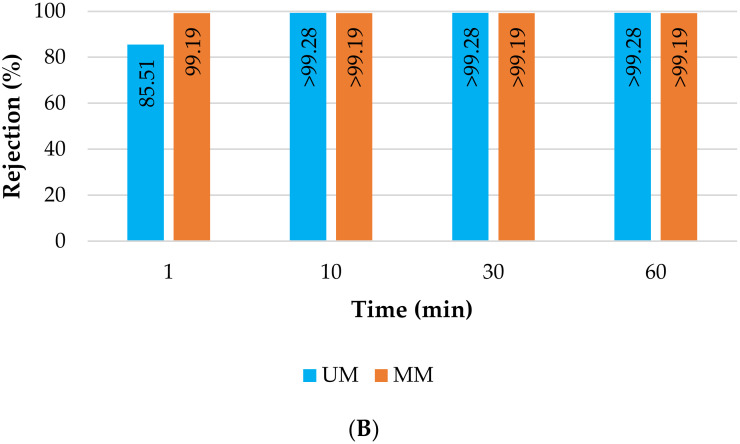
Rejection percentage of total coliforms (**A**) and *Escherichia coli* (**B**) from the untreated surface water samples with unmodified (UM) and modified membrane (MM) after 1, 10, 30, and 60 min of filtration. Values marked “>” indicate that the target microorganisms were not detected in the treated water samples.

Figure A3 presents the percent rejection results for total coliforms and *E. coli* obtained at different time points. In the early stages of the experiments the low contamination of the permeate can be explained by the membranes’ maximum Feret diameters of 5.5 µm, as measured at various zones of the membranes. The higher percentage of rejections obtained after 10 min of filtration may be due to fouling at the membranes’ surface (Figure A3).

#### 3.2.2. Direct Photolysis Treatment

Figure 4 shows the percent inactivation of total coliforms and *E. coli*, as well as total coliforms and *E. coli* resistant to various antibiotics (ciprofloxacin, levofloxacin, imipenem, ampicillin, and streptomycin) obtained after one hour of direct photolysis exposure to the LED panels that emit at 265 nm and the low-pressure mercury lamps (LP-UV).

**Figure A4:**
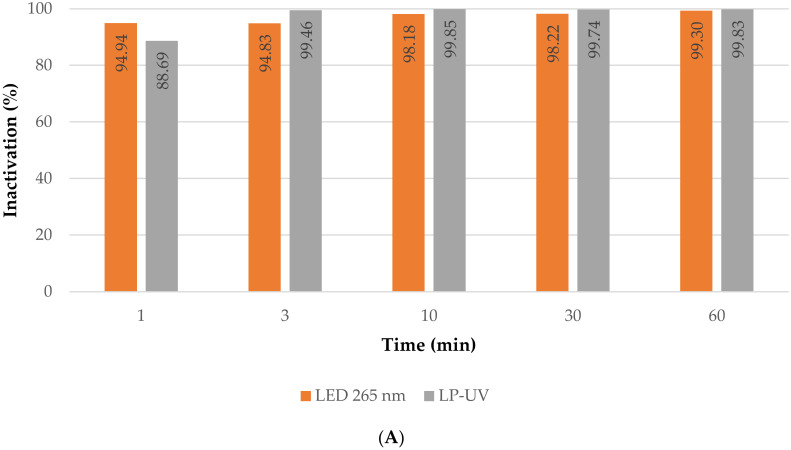

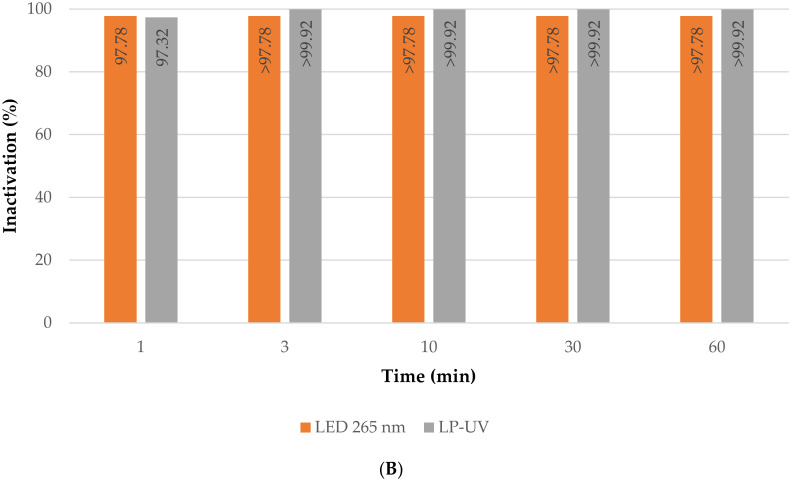
Inactivation percentage of total coliforms (**A**) and *Escherichia coli* (**B**) from the untreated surface water samples with LEDs emitting at 265 nm and low-pressure ultraviolet lamps (LP-UV) after 1, 3, 10, 30, and 60 min. Values marked “>” indicate that the target microorganisms were not detected in the treated water samples.

The inactivation performance obtained using the LP-UV lamps and the LED panels that emit light at 265 nm was extremely efficient (Figure 4 and Figure A4). These results were expected due to the peak absorption of DNA being around 260 nm [61]. Several studies have focused on the inactivation of microorganisms using LP-UV lamp and LED systems [62,63,64,65,66,67]. Li et al. [65] demonstrated that LEDs emitting at 265 nm were more efficient at *E. coli* inactivation than LP UV lamps. In this study different real water samples were used in the various assays, so a direct comparison between the different light sources cannot be made. However, the results obtained show that both light sources were extremely effective at coping with total coliforms resistant to several antibiotics, since the target microorganisms were not detected in the treated water samples. Several authors have reported that antibiotic-resistant bacteria are less sensitive to UV irradiation [68,69,70,71]. It is therefore important to test the treatment of resistant bacteria by different light sources, the combined treatment by membrane filtration and UV-C light, and photocatalysis. Zhang et al. [69] reported that multiple-antibiotic-resistant *E. coli* were more resistant at low UV doses and required a higher UV dose to enter the tailing phase compared with antibiotic-sensitive *E. coli*.

#### 3.2.3. Combined Treatment

One major drawback of filtration processes is the production of a concentrate retentate [17]. Coupling UV photolysis with membrane filtration using an unmodified or a modified membrane allows the retention and effective inactivation of microorganisms [17,21,22].

The results shown in Figure 5 depict the feed treatment percentage of total coliforms and *E. coli*, and total coliforms and *E. coli* resistant to various antibiotics (ciprofloxacin, levofloxacin, imipenem, ampicillin, and streptomycin). As expected, in line with previous results for individual treatment obtained for the antibiotic-resistant bacteria (Figure 3 and Figure 4) and previous results reported for water quality indicator bacteria [21,22], the combined treatment provided extremely effective retention and treatment of the total coliforms and *E. coli*, as well as the antibiotic-resistant bacteria, from the feed stream (Figure 5).

To clarify whether the light sources inactivated the bacteria retained on the surface of the membranes, additional experiments were carried out. Hybrid reactor tests were performed with 10 L of untreated surface water recirculated for one hour, during which period the microorganisms were retained by the membranes. After the recirculation time, the membranes used were carefully wiped and cleaned using 400 mL of sterile distilled water to remove and quantify the retained bacteria. The inactivation of antibiotic-resistant bacteria adsorbed by the membranes is shown in Figure 6.

In agreement with the results obtained in the direct photolysis experiments, the LP-UV and the UV-C LED panels emitting at 265 nm achieved extremely high inactivation levels. The inactivation of total coliforms was 96.2% and 99.5%, respectively and for *E. coli* were 96.0% and >99.9%, respectively. Furthermore, the combined treatment with UV-A LEDs that emit light at 385 nm and the modified membrane with titanium dioxide and copper achieved inactivation levels higher than 76.9% for total coliforms and 40.8% for *E. coli*. For the antibiotic-resistant total coliforms and *E. coli*, a similar trend of inactivation was observed.

## 4. Conclusions

This study highlights the frequent detection of total coliforms and *E. coli* resistant to ampicillin, streptomycin, ciprofloxacin, and levofloxacin in river water samples. Several real water samples were collected to test the effectiveness of different treatment processes: membrane filtration, direct photolysis, and a combined treatment. Without any pre-treatment, all the tested treatment options can be expected to effectively remove antibiotic-resistant bacteria present at occurrence levels in surface water.

The membranes modified with titanium dioxide and copper presented no significant improvement compared with the unmodified membranes for retention of the target bacteria. Hence, if the system had to be replicated on a larger scale, with this purpose in mind (retention of bacteria), it would be beneficial to use the unmodified membranes due to their lower cost and higher water throughput. The combined treatment using unmodified membranes and UV-C light sources (low pressure mercury lamps and LED panels that emit at 265 nm) proved extremely effective at retaining and inactivating antibiotic-resistant water quality indicators.

The modified membranes may prove to have advantages if the combined treatment is applied using UV-A LEDs or solar light.

Various treatment solutions can be proposed such as: (a) using low-pressure mercury lamps and LEDs that emit at 265 nm as a tertiary treatment to achieve inactivation in water treatment plants; (b) using compact systems with several membranes intercalated by UV-C light sources in sequence for water treatment in large-scale plants; and (c) smaller point-of-use systems with the modified membranes activated by solar light.

## Figures and Tables

**Table 1:** pH and content in solids of the untreated surface water samples collected in this study.

Parameters	Average and Standard Deviation
pH	7.2 ± 0.6
Total solids (g/L)	34.6 ± 3.6
Total suspended solids (g/L)	1.0 ± 0.3
Total dissolved solids (g/L)	33.6 ± 3.6

## Data Availability

Not applicable.

## Appendix A

**Supporting Information Regarding the Quantification of Antibiotic-Resistant Bacteria.**

The concentration of antibiotics used was based on the minimal inhibitory concentration (MIC) breakpoints levels for Enterobacteriaceae, documented by Clinical and Laboratory Standards Institute (CLSI) (M100—Performance Standards for Antimicrobial Susceptibility Testing, 28th edition, January 2018) and in the literature [42,43]. Stock solutions of ciprofloxacin (Acros Organics, Geel – Belgium), levofloxacin (Sigma Aldrich, St. Louis, MO, USA), imipenem (Sigma Aldrich, St. Louis, MO, USA), ampicillin (nzytech, Lisbon, Portugal), and streptomycin (Sigma Aldrich, St. Louis, MO, USA) were prepared. A volume of stock antimicrobial solution was added to 100 mL of the water sample to achieve the required final concentrations of ciprofloxacin (4 µg/mL), levofloxacin (8 µg/mL), imipenem (4 µg/mL), ampicillin (32 µg/mL), and streptomycin (64 µg/mL).

**Table A1:** Total coliforms and *Escherichia coli* (MPN/100 mL) and total solids, total suspended solids, and total dissolved solids (g/L) present in the surface water samples used in the filtration experiments using the unmodified and modified membranes.

	Total Coliforms (MPN/100 mL)	*Escherichia coli* (MPN/100 mL)	Total Solids (g/L)	Total Suspended Solids (g/L)	Total Dissolvel Solids (g/L)
Unmodified membrane	376	138	32	1	31
Modified membrane	266	124	41	1	40
